# Deletion of IL-33R attenuates VEGF expression and enhances necrosis in mammary carcinoma

**DOI:** 10.18632/oncotarget.7635

**Published:** 2016-02-23

**Authors:** Milos Z. Milosavljevic, Ivan P. Jovanovic, Nada N. Pejnovic, Slobodanka L. J. Mitrovic, Nebojsa N. Arsenijevic, Bojana J. Simovic Markovic, Miodrag L. Lukic

**Affiliations:** ^1^ Department of Pathology, University Medical Center Kragujevac, Kragujevac, Serbia; ^2^ Center for Molecular Medicine and Stem Cell Research, Faculty of Medical Sciences, University of Kragujevac, Kragujevac, Serbia

**Keywords:** breast neoplasm, IL-33, IL-33R, neoangiogenesis, necrosis

## Abstract

Interleukin-33 (IL-33)/IL-33 receptor (IL-33R, ST2) signaling pathway promotes mammary cancer growth and metastasis by inhibiting anti-tumor immunity. However, the role of IL-33/IL-33R axis in neoangiogenesis and tumor necrosis is not elucidated. Therefore, the aim of this study was to investigate the role of IL-33/IL-33R axis in mammary tumor necrosis. Deletion of IL-33R (ST2) gene in BALB/c mice enhanced tumor necrosis and attenuated tumor growth in 4T1 breast cancer model, which was associated with markedly decreased expression of vascular endothelial growth factor (VEGF) and IL-33 in mammary tumor cells. We next analyzed IL-33, IL-33R and VEGF expression and microvascular density (MVD) in breast tumors from 40 female patients with absent or present tumor necrosis. We found significantly higher expression of IL-33, IL-33R and VEGF in breast cancer tissues with absent tumor necrosis. Both, IL-33 and IL-33R expression correlated with VEGF expression in tumor cells. Further, VEGF expression positively correlated with MVD in perinecrotic zone. Taking together, our data indicate that IL-33/IL-33R pathway is critically involved in mammary tumor growth by facilitating expression of pro-angiogenic VEGF in tumor cells and attenuating tumor necrosis. These data add an unidentified mechanism by which IL-33/IL-33R axis facilitates tumor growth.

## INTRODUCTION

Breast cancer is the second most common cancer in the world and the most frequent cancer among women [1]. Although multidisciplinary approach improved overall survival and quality of life of breast cancer patients, identification of new prognostic markers and therapeutic modalities are needed.

Chronic ischemia followed by hypoxia can cause necrosis of tumor cells [2]. Tumor necrosis is associated with natural history of mammary carcinoma [3, 4]. Some studies have shown that tumor necrosis is a poor prognostic factor which is associated with high tumor proliferative activity [5, 6]. However, recent studies suggest that tumor necrosis could be beneficial as a favorable outcome of anti-cancer therapy and may enhance anti-tumor immune response [4, 7-12].

Necrotic cells release immunoregulatory cytokines, including interleukin-33 (IL-33) [13, 14], a member of the interleukin-1 (IL-1) family of cytokines [15]. IL-33 is dual function protein with roles as a nuclear factor and a classical cytokine [16] and functions as a prototypic „alarmin” [17]. As a cytokine, IL-33 binds a heterodimeric receptor complex comprised of IL-1 receptor-like 1 (IL1RL1; also referred to as ST2L) and its coreceptor, IL-1 receptor accessory protein (IL- 1RAcp), which regulates inflammatory gene expression through NF-κB (nuclear factor kappa-light-chain-enhancer of activated B cells) and MAPK (Mitogen-activated protein kinases) signaling pathways [18, 19]. IL-33 participates in many immune disorders exerting pro-, or anti-inflammatory roles [20-25].

The role of IL-33 in cancer is still unclear. We have previously shown that deletion of IL-33/IL-33R axis facilitated anti-tumor immunity which led to attenuated tumor growth and metastasis in experimental mammary carcinoma [18, 26]. In addition, we demonstrated that IL-33/IL-33R axis promoted breast cancer growth and metastases by facilitating intratumoral accumulation of immunosuppressive and type 2 innate lymphoid cells [27]. However, it was reported that transfection of IL-33 in tumor cells attenuated development of metastasis in B16 melanoma and in Lewis lung carcinoma metastatic models in mice [28]. Sun et al [29] reported that elevated serum levels of IL-33 in patients with gastric cancer correlate with disease severity and progression. Serum levels of IL-33 are a diagnostic and prognostic marker in non-small cell lung cancer [30]. Patients with breast cancer have higher serum levels of IL-33 compared to patients with benign breast diseases and IL-33 expression was more pronounced in breast tumors than in normal breast tissue [31].

Here, we report that IL-33/IL-33R signaling inhibits tumor necrosis in mouse mammary carcinoma. Deletion of IL-33R gene in mice favors tumor necrosis and decreases expression of IL-33 and VEGF in mammary tumors. Further, in breast tumors from patients with similar clinicopathologic characteristics, the presence of tumor necrosis was associated with lower expression of IL-33, IL-33R and VEGF. Our findings reveal a novel role for IL-33/IL-33R pathway in breast cancer neoangiogenesis and necrosis.

## RESULTS

### IL-33R deletion enhances tumor necrosis and attenuates mammary tumor growth in mice

**Figure 1:**
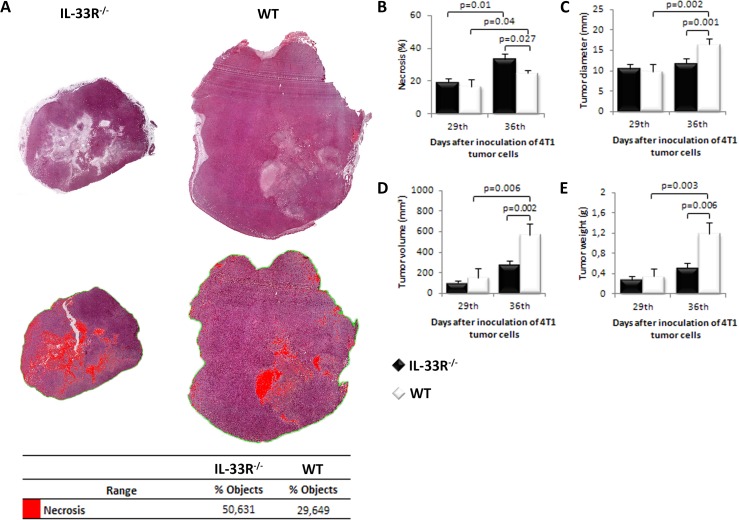
Genetic deletion of IL-33R favors tumor necrosis and attenuates tumor growth in 4T1 breast carcinoma in mice **A.** Mammary tumor necrosis was analyzed using Image Pro Plus software (v. 6.0.0.206) and presented as a percentage of necrotic area (red pixels) compared to the area of tumor tissue (ROI). **B.** The presence of IL-33R reduced tumor necrosis **C.** Tumor diameter **D.** Tumor volume E. Tumor weight in WT and IL-33R^−/−^ mice. Statistical significance was tested by Mann-Whitney Rank Sum test.

In order to investigate the role of IL-33/IL-33R axis in tumor necrosis we used 4T1 metastatic breast cancer model in IL-33R-deficient BALB/c mice. Mice were sacrificed on days 29 or 36 after tumor inoculation. We first investigated the extent of necrosis in primary mammary tumors at days 29 and 36. As shown in Figure 1A and 1B the extent of tumor necrosis appear to be higher in IL-33R^−/−^ mice with statistically significant larger extent of necrosis on day 36 after tumor challenge (*p* = 0.027; Figure 1B). In order to compare the degree of tumor necrosis with other parameters of tumor expansion, we measured tumor diameter, volume and weight. These parameters were evaluated on day 36 and indicated significantly faster tumor growth in WT mice which had less extensive tumor necrosis (all *p* < 0.01; Figure 1C, 1D and 1E).

### IL-33 expression in mammary tumor cells correlates with tumor growth in WT, but not in IL-33R^−/−^ mice

**Figure 2:**
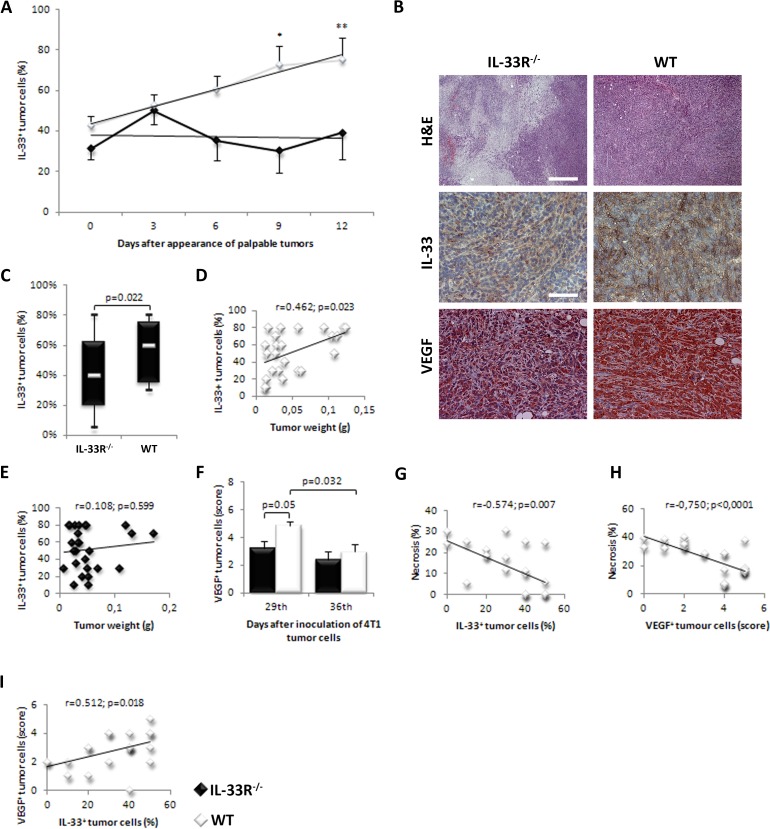
IL-33 and VEGF expression in mammary carcinoma is lower in IL-33R deficient mice **A.** IL-33 expression in mammary tumor cells significantly increased over time in WT mice (*p = 0.019, **p = 0.003). **B.** H&E staining (original magnifications: x100, scale bar: 200μm) and immunohistochemical expression of IL-33 (Upper panel) and VEGF (Lower panel) (original magnifications: x200, scale bar: 100μm) in tumor tissue in IL-33R^−/−^ and WT mice, representative tissue sections. **C.** Percentage of cytoplasmic expression of IL-33 and difference in percentage of cytoplasmic expression of IL-33 in tumor cells between IL-33R^−/−^ and WT mice. **D.** Correlation between tumor weight and IL-33 expression in tumor cells in WT and IL-33R^−/−^ mice. **E.**, **F.** Expression of VEGF in tumor tissue at 29th and 36th day after inoculation of 4T1 cells in IL-33R^−/−^ and WT mice. **G.** Correlation between the extent of tumor necrosis and IL-33 expression in tumor cells in WT mice. **H.** Correlation between the extent of tumor necrosis and VEGF expression in tumor cells in WT mice. **I.** Correlation between expression of VEGF and IL-33 in tumors in WT mice. Statistical significance was tested by Mann-Whitney Rank Sum test and by Spearman correlation coefficient.

IL-33 expression in mammary tumor cells significantly increased over time in WT mice (Figure 2A and 2B - upper panels), but not in IL-33R^−/−^ mice (Figure 2A), the findings that reflect positive feedback mechanism in IL-33/IL-33R axis. Further, overall IL-33 expression in tumor tissue during the observed period of time was significantly higher in WT mice (*p* = 0.022; Figure 2C). Immunohistochemical analyses revealed strong correlation between the expression of IL-33 in tumor tissue and tumor weight in WT mice (*r* = 0.462; *p* = 0.023; Figure 2D), which was not observed in IL-33R^−/−^ mice (*r* = 0.108; *p* = 0.599; Figure 2E).

### IL-33 and VEGF expression negatively correlate with mammary tumor necrosis

Deletion of IL-33R attenuated VEGF expression in mammary tumor cells with statistically significant difference at day 29 after tumor inoculation compared to WT mice (*p* = 0.05; Figure 2F and 2B - lower panel). IL-33 expression negatively correlated with the size of tumor necrosis at day 36 after tumor challenge in WT mice (*r* = −0.574; *p* = 0.007; Figure 2G). In line with this finding, VEGF score also negatively correlated with the size of tumor necrosis at day 36 after tumor challenge in WT mice (*r* = −0.750; *p* < 0.0001; Figure 2H), but not in IL-33R^−/−^ mice (data not shown). Finally, IL-33 expression positively correlated with VEGF score in tumor cells in WT mice (*r* = 0.512; *p* = 0.018; Figure 2I).

### Expression of IL33, IL-33R and VEGF in human breast carcinoma cells inversely correlate with tumor necrosis

In 40 female patients with invasive breast carcinoma we analyzed IL-33, IL-33R and VEGF expression in tumor cells in order to investigate the relevance of experimental findings in mice for corresponding human pathology. Clinicopathologic characteristics of patients are presented in Table 1. Breast tumors analyzed in this study were of similar size and classified into T2 and T3 stages based on the American Joint Committee on Cancer (AJCC) TNM system [32] (Table 1).

**Table 1:** Characteristics of 40 patients with invasive ductal breast carcinoma

		Necrosis		
Variables		Absent N (%)	Present N (%)	*p* value
Enrolled patients		20 (50.00%)	20 (50.00%)	
Age (mean ± SEM)		56.7 ± 2.23	58.1 ± 2.56	0.704
Size (mean ± SEM)		27.7 ± 1.71	36.3 ± 3.07	0.126
AJCC stage	I+II	14 (35.00%)	10 (25.00%)	0.333
	III	6 (15.00%)	10 (10.00%)	

AJCC: American Joint Committee on Cancer, 7^th^ Ed., 2010

In order to correlate tumor necrosis with IL-33, IL-33R and VEGF expression, patients were divided into two groups based on the presence (20 patients) or absence (20 patients) of necrotic fields in breast tumor tissue [33]. There were no differences in age, tumor size and AJCC stage between two groups of patients with present or absent tumor necrosis (Table 1).

**Figure 3:**
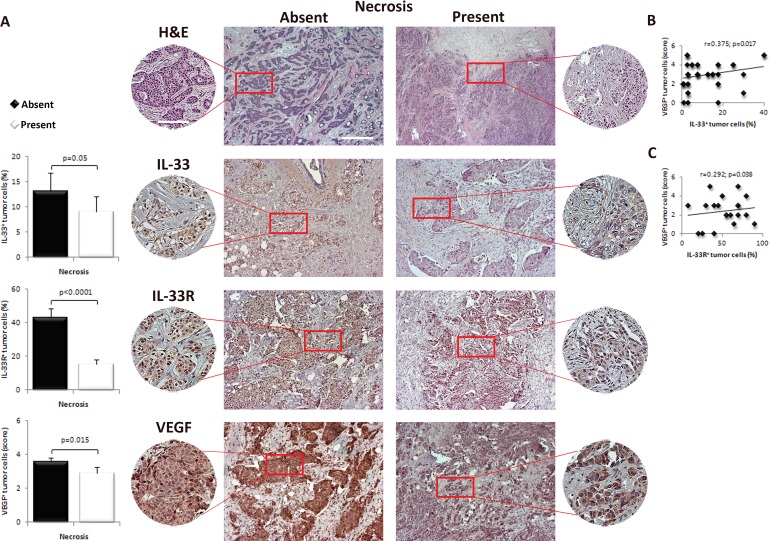
IL-33, IL-33R and VEGF expression in human breast carcinoma with present or absent tumor necrosis **A.** Representative images of human mammary carcinoma with and without necrosis (H&E, original magnification: x100, scale bar: 200μm; x200, scale bar: 100μm). IL-33, IL-33R and VEGF expression was evaluated by immunohistochemistry for each patient (original magnification: x100, scale bar: 200μm; x200, scale bar: 100μm). The difference in the expression of immunohistochemical markers was examined between tumors with and without necrosis. **B.**, **C.** Correlation between IL-33 and IL-33R with VEGF expression in tumor cells in human mammary carcinoma tissues. Statistical significance was tested by Mann-Whitney Rank Sum test, independent samples t-test or Spearman correlation coefficient.

We analyzed expression levels of IL-33, IL-33R and VEGF in tumor cells in breast cancer tissues in two groups of patients. Representative images of immunostained breast tumor tissues with these markers are shown in Figure 3. IL-33 and IL-33R expression in tumor cells was lower in breast tumors with necrosis (*p* = 0.05 and *p* < 0.0001, respectively; Figure 3A). Similarly, expression of VEGF in tumor cells was lower in tumors with necrosis (*p* = 0.015; Figure 3A). In mammary tumor cells both IL-33 and IL-33R expression positively correlated with VEGF expression (*r* = 0.375; *p* = 0.017; *r* = 0.292; *p* = 0.038, respectively; Figure 3B and 3C).

### VEGF expression positively correlates with MVD in perinecrotic human breast cancer tissue

**Figure 4:**
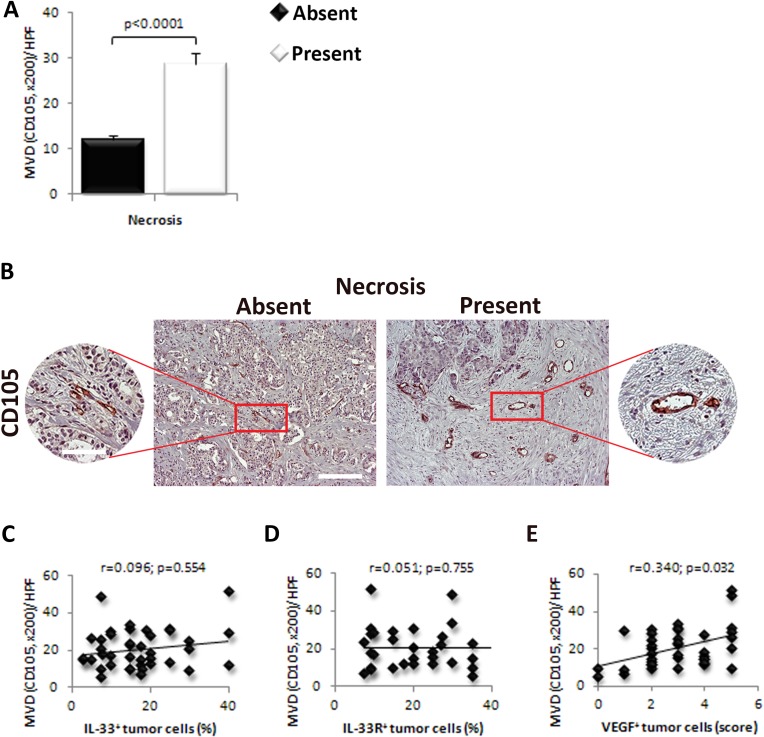
VEGF expression in tumor cells correlate with the expression of IL-33 and IL-33R and MVD in human breast cancer **A.** The difference in the MVD was examined between tumors with present or absent necrosis. **B.** Representative images of CD105 immunostaining in human breast carcinoma with and without necrosis (original magnification: x100, scale bar: 200μm; x200, scale bar: 100μm). **C.**, **D.** Correlation between the expression of IL-33 and IL-33R in perinecrotic tumor cells with MVD in human breast carcinoma. **E.** Correlation between the expression of VEGF in perinecrotic tumor cells with MVD in human breast carcinoma. Statistical significance was tested by Mann-Whitney Rank Sum test or Spearman correlation coefficient.

The MVD was higher in tumors with necrosis in comparison to those without necrotic fields (*p* < 0.0001; Figure 4A and 4B). Blood vessels were grouped in clusters in tumor tissues mainly around the necrotic zone (Figure 4A and 4B). Next, we analyzed the association of IL-33, IL-33R and VEGF expression with MVD in perinecrotic breast cancer tissue. We did not find significant correlation between the expression of IL-33 (*r* = 0.096; *p* = 0.554) and IL-33R (*r* = 0.051; *p* = 0.755) in perinecrotic zone and MVD in tumor tissue Figure 4C and 4D). However, there was statistically significant positive correlation between the expression of VEGF in tumor cells around the necrotic zone and MVD (*r* = 0.340; *p* = 0.032; Figure 4E).

## DISCUSSION

In this study, we show that deletion of a receptor for immunoregulatory IL-33 promotes mammary tumor necrosis in mice (Figure 1A and 1B). This finding appears to be a novel mechanism by which IL-33 promotes tumorogenesis. It has been previously shown that IL-33/IL-33R axis suppressed innate anti-tumor immunity and apparently favored neoangiogenesis [26, 27]. Here, we show that enhanced tumor necrosis in IL-33R^−/−^ mice correlates to lower expression of angiogenic factors IL-33 and VEGF (Figure 2A-2C and 2F-2H) and is associated with slower tumor growth (Figure 1A). The significance of tumor necrosis in tumor expansion and metastasis is still debatable [33, 34]. Although the appearance of necrosis in the natural history of breast carcinoma was associated with unfavorable outcome, therapeutically induced tumor necrosis may be beneficial [4-12]. Thus, attenuation of IL-33 may stimulate innate antitumor immune response, but also may promote tumor necrosis which is associated with the lack of VEGF [35, 36]. In addition, the presence of tumor necrosis may favor innate anti-tumor immune response [2, 37], as necrotic cells facilitate maturation of antigen-presenting cells [38].

In this study, the findings in human breast cancer tissues were in general agreement with findings in experimental mammary carcinoma. When patient groups were equalized in relation to age, tumor size, status of axillary lymph nodes and AJCC stage of tumors we found that the level of tumor necrosis correlated with lower expression of IL-33, IL-33R and VEGF. Further, IL-33 and IL-33R expression positively correlated with VEGF expression (Figure 3B and 3C).

IL-33, like VEGF, facilitates proliferation and migration of endothelial cells, as well as junctional permeability and vessel leakage, *in vitro* [39]. These roles of IL-33 seem not to be mediated through VEGF [39]. At present, there is no consensus which of these two pro-angiogenic factors is more responsible for tumor neoangiogenesis.

Expression of IL-33 is positively associated with VEGF expression, in animal and human breast cancer cells (Figure 2I and 3B). The presence of IL-33R increases the expression of IL-33 (Figure 2A). In IL-33R^−/−^ mice we detected lower expression of VEGF in tumor cells (Figure 2F), suggesting that IL-33/IL-33R signaling is associated with expression of VEGF (Figure 2F and 2I).

Theoharides et al [40] suggested that IL-33 directly facilitated VEGF expression and secretion in primed mast cells. Study by Stojkovic et al [41] showed proangiogenic role of IL-33 by inducing endothelial angiogenic activity via IL-33R expressed on endothelial cells, the effects that were not mediated by VEGF, but by increased urokinase-type plasminogen activator activity.

Thus, it is likely that blocking of IL-33/IL-33R signaling on its own and by attenuating VEGF expression in tumor cells and subsequent neoangiogenesis promote tumor necrosis.

Interestingly, we found increased MVD in human mammary carcinoma tissue with detectable necrosis (Figure 4A and 4B). There was a high-grade MVD in residual tumor tissue around the necrotic zone (Figure 4A and 4B). There was no significant correlation between the expression of IL-33 and IL-33R and MVD (Figure 4C and 4D), while VEGF expression in tumor cells positively correlated with MVD in perinecrotic zone (Figure 4E).

It should be noted that other members of IL-1 family exert significant effect in tumor angiogenesis [42-44]. On the basis of these observations we suggest that IL-33 released by necrotic cells may facilitate VEGF expression on nearby tumor cells, which could lead to enhanced angiogenesis as demonstrated by high-grade MVD in perinecrotic zone in human breast cancer tissue.

IL-33 is very much like IL-1 in that both IL-1β and IL-33 are proangiogenic and control the production of VEGF. IL-1β increases expression of VEGF and its receptors on endothelial cells and acts together with VEGF in promoting tumor mediated angiogenesis. Thus, the neutralization of IL-1β reduced tumor growth and the tumor-induced angiogenesis [42-44]. As IL-1 and IL-33 both use IL-1R3 (IL-1RAcP), it may be speculated that anti-IL-1R3 antibodies may reduce angiogenesis and increase tumor necrosis in breast cancer due to loss of vascular supply. This concept is supported by recent studies showing high expression of IL-1R3 in acute myelogenous leukemia [45]. Thus, this study revealed an additional mechanism by which IL-33/IL-33R pathway may be involved in tumorigenesis and provide rationale for blocking IL-33 as a therapeutic modality in human breast carcinoma.

## MATERIALS AND METHODS

### Ethical approvals

The study was conducted at the Department of Pathology, University Medical Center Kragujevac, Serbia and the Center for Molecular Medicine and Stem Cell Research, Faculty of Medical Sciences, University of Kragujevac, Serbia.

Experimental animals used in this study received human care and all experiments were approved by and conducted in accordance with, the Guidelines of the Animal Ethics Committee of the Faculty of Medical Sciences, University of Kragujevac, Serbia. Patients included in the study gave their informed consent and the research project was approved by the Ethics Committees of the University Medical Center Kragujevac, Serbia. Additionally, adherence was made to the Principle of Good Clinical Practice and the Declaration of Helsinki at all times.

### Mice

We used 10-weeks-old female wild type BALB/c (WT) and ST2 knockout (IL-33R^−/−^) mice on BALB/c background (generated as described previously by Townsend et al) [46]. Mice were maintained in animal breeding facilities of the Faculty of Medical Sciences, University of Kragujevac, Serbia. Mice were housed in a temperature-controlled environment with a 12-hour light-dark cycle and were administered standard laboratory chow and water *ad libitum*.

### 4T1 tumor cell line

4T1 mammary carcinoma cell line, syngeneic to the BALB/c background, was obtained from American Type Culture Collection (ATCC, USA). The cells were cultured in Dulbecco's Modified Eagle Medium (DMEM) containing 10% heat-inactivated fetal bovine serum (FBS), 2 mmol/L L-glutamine, 1mmol/L penicillin-streptomycin, 1mmol/L mixed nonessential amino acids (Sigma-Aldrich chemical, Munich, Germany) (complete growth medium), in a humidified incubator at 37°C with 5% CO_2_. Experiments were conducted on low passage cells. The number of viable tumor cells was determined by trypan blue exclusion and only cell suspensions with ≥95% viable cells were used.

Mice were inoculated with 5×10^4^ 4T1 tumor cells/50μl phosphate-buffered saline (PBS, Invitrogen, USA) orthotopically into the fourth mammary fat-pad, as described previously [47]. Mice were sacrificed with ether 29 and 36 days after tumor inoculation and tumors excised and weighed. In order to follow up expression of IL-33 between WT and IL-33R^−/−^ groups, mice were sacrificed at every third day after tumor appearance.

### Estimation of *in vivo* 4T1 tumor growth

After the extirpation, the weight of the primary 4T1 mammary tumors was determined using analytical balance. The size of primary tumors was assessed morphometrically using electronic calipers in two dimensions and tumor volumes (mm3) were calculated as *L x W^2^/2*, where L represents the major axis of the tumor and W represents minor axis [26].

### Quantification of tumor necrosis

Tumor tissue sections were photographed with individual images in the worm manner at a magnification x 200. Images were embedded in the mosaic using Adobe Photoshop CS6 software *(Adobe Systems Incorporated, San Jose, CA, USA)* package. In this way we obtained microscopic images of the entire cross section of the tumor. The generated mosaics were analyzed using Image Pro Plus software version 6.0.0.206 *(Media Cybernetics, Inc., Rockville, MD, USA)*. In the first step we determined regions of interest (ROI) confining tumor tissue relative to the background (the green line). Quantification of necrosis was estimated selecting the range of colors to match the necrotic tissue (red pixels). The measurement results are displayed as a percentage share of necrotic fields in ROI [10].

### Human breast cancer tissues

Paraffin embedded breast tissue sections were obtained from 40 female patients with diagnosis of invasive ductal carcinoma, between January 2003 and March 2005, registered in the University Medical Center Kragujevac, Serbia. Each diagnosis was confirmed by a pathologist, on hematoxylin and eosin stained slides, using standard histopathologic criteria. Diagnosis was established according to World Health Organization (WHO) criteria on the ground of post-operative histopathological examination [3]. Patients with breast cancer were classified into two groups based on the presence of necrosis (absent or present) [2].

### Immunohistochemical detection of VEGF, IL-33 and IL-33R in mouse and human tissues

Paraffin-embedded primary 4T1 tumor tissue sections and surgically removed human breast cancers were fixed in 10% neutral buffered formalin and embedded in paraffin using standard pathological protocols. Immunohistochemistry was performed on a single representative block from each case, as previously described [48]. Antigenic retrieval was processed by submerging the sample in 10mM citrate buffer (pH 6) or commercial buffer (10mM EDTA Buffer for Heat-Induced Epitope Retrieval (pH8), AP-9004-125, Thermo Scientific, Waltham, MA, USA) and microwaving for 20 minutes at 96°C. Primary polyclonal antibodies were directed against IL-33R (human: 1:500, PA5-20077, Thermo Scientific, Waltham, MA, USA), IL-33 antigen (mouse/human: 1:200 dilution, sc-98660, Santa Cruz Biotechnology, Santa Cruz, CA, USA), and VEGF (mouse: 1:120 dilution, ab46154, Abcam, Cambridge, UK; human: 1:100 dilution, RB-9031-P0, Thermo Scientific, Waltham, MA, USA). Tissue sections were incubated with appropriate primary antibody and commercial biotinylated secondary anti-immunoglobulin, at room temperature, according to the manufacturer's instructions (UltraVision LP Large Volume Detection System: HRP Polymer (Ready-To-Use), TL-125-HL, Thermo Scientific, Waltham, MA, USA). Specificity was verified by negative control reactions without primary antibody, as well as appropriate cytoplasmic reactions for each antigen in positive control tissues. The slides were examined and analyzed at x100, x200 and x400 magnification, using conventional light microscopy (Axioskop 40, Carl Zeiss, Oberkochen, Baden-Württemberg, Germany).

The VEGF, IL-33 and IL-33R stained sections were assessed semiquantitatively by two pathologists. For VEGF expression, immunohistochemical reactions were assessed in areas of invasive carcinoma. VEGF expression was evaluated according to scoring system described by Rydén et al [49] for VEGF interpretation in invasive breast carcinoma. VEGF protein expression was observed in the cytoplasm of tumor cells, and the immunohistochemical score was calculated according to the percentage of positively stained cells. The quantity score ranges from 0 to 5: 0 = lack of staining; 1 = ≤1% cells stained; 2 = 1-10% cells stained; 3 = 10-50% cells stained; 4 = 50-90% cells stained and 5 ≥ 90% cells stained. Immunohistochemical expression of IL-33 and IL-33R in tumor cells was independently quantified as the percentage of positive cells out of the total number of evaluated cells. Analyses were performed by counting 5 non overlapping microscopic fields at an x400 magnification.

### Evaluation of MVD in human breast cancer tissues

For the detection of microvessels, anti-CD105 monoclonal antibody (human: Ready-To-Use, RB-9291-R7, Thermo Scientific, Waltham, MA, USA) was applied followed by secondary antibody, as previously described [48]. Single endothelial cells or clusters of endothelial cells positive for CD-105 were considered as a microvessel, by two pathologists [50]. At first, slides were examined at an original magnification of x40. Three „hot spots“ (areas with the highest microvessel density) from each slide were identified and these areas were photographed by a digital camera (AxioCam ICc1, Carl Zeiss, Oberkochen, Baden-Württemberg, Germany) at an original magnification of x200. The area of this histological field was 0,704μm^2^. MVD (microvessel/HPF) and number of microvessels evaluated according to Weidner et al [51]. MVD of the specimen was estimated as a mean of MVD in three histological fields.

### Statistical analysis

The SPSS statistical software package version 20.0 (SPSS Inc., Chicago, IL, USA) was used for analyses of the data. The normality of distribution was tested by Kolmogorov-Smirnov test. The two-tailed Student's t test, Fisher's Exact Test or nonparametric Mann-Whitney Rank Sum test were used where appropriate. *Pearson*'s or Spearman's correlation coefficient was used to test the correlations between two variables. All reported *P* values were 2-sided and *p* < 0.05 was considered statistically significant and highly significantly different when *p* < 0.01. The data are presented as the mean ± standard error of the mean.
